# International Congress

**Published:** 1887-08

**Authors:** 


### International Congress.

### FUNDS FOR THE CONGRESS.

*To the Medical Profession:*

The Local Committee of Arrangements have the pleasure to announce to their American brethren that the widespread desire to attend the Congress is such that the amount of money for the reception and entertainment heretofore deemed sufficient will be entirely insufficient to provide for the large number that will be in attendance. They are therefore constrained to appeal to their brethren throughout the country for additional subscriptions to the entertainment fund. They feel that to their patriotic countrymen it is only necessary for the fact to be stated in order to secure the sending of such liberal contributions as will ensure the entire success of the social features of this great international gathering on a scale commensurate with its dignity and importance. Let all Americans come to the front and ensure to *all* the foreign members the full measure of the hospitality of free America. Contributions should be immediately forwarded to Dr. C. W. Franzonni, member of the Finance Committee for the District of Columbia.

By order of the Local Committee of Arrangements.

C. H. A. Kleinschmidt, *Secretary.*

Washington, D. C., July 30, 1887.

[Medical editors, friendly to the good cause, will confer a favor by publishing this appeal.—Ed.]

### EXCURSION RATES TO CALIFORNIA.

Dr. Liston H. Montgomery:

*Dear Sir:*—I have been repeatedly asked by gentlemen of the medical profession, who will attend the International Congress at Washington, in September next, what would be the cost of a trip to California. In reply I wish to say that an excursion to California will be had after the adjournment of the Congress. Parties can take the route via New Orleans, returning via the Central Pacific Railroad, thus enabling the excursionists to see a large part of the country at a cost of a little less than the fare one way. You may inform all who inquire that a low rate will be made. This trip would be especially beneficial to the physicians from Europe, and also to those who have never visited California.

Respectfully,

H. M. Van Arman, T. P. A. S. P. Co.

Chicago, July 28, 1887.

Note.—Dr. L. H. Montgomery assures us that at a later date than the above he was authorized by Mr. Van Arman to state that the round trip either from Washington or Niagara to California and return would be made at about $90, or less than the usual railroad fare one way; and that the Pullman sleeping cars for the trip would be $14 in addition. These terms are offered alike to the foreign and American members of the Congress. Tickets will be good for 60 or 90 days.

SECRETARYSHIP DENTAL AND ORAL SURGERY.

*Mr. Editor:*—Will you please give notice that, as I am unable to continue the work of the Secretaryship of the Dental and Oral Section of the International Medical Congres, Dr. A. M. Dudley, of Salem, Mass., has kindly consented to act. All communications should henceforward be addressed to him.

Respectfully yours,

E. A. Bogue.

### Advance Registration for the Congress.

Under the head of International Medical Congress, in another column, will be found the form of *application* in blank, for members of the regular medical profession, in the United States, to *fill correctly* and send, or present in person, to the Chairman of the Committee on Registration, J. M. Toner, M.D., 615 Louisiana Avenue, Washington, D. C., with the membership fee, $10, and each person sending the application for registration in advance, by mail, should enclose a postage stamp to pay for return receipt or ticket of admission in return.

### Railroad Rates to Washington and Old Point Comfort.

EDITOR REGISTER:

*Dear Sir*—For delegates who wish to attend at Old Point Comfort before the Washington meeting we will furnish tickets from Cincinnati to Old Point Comfort, via C. & O.; Old Point to Washington via Potomac Steamboat Co.; back to Cincinnati, all rail, via. C. & O. $21.00.

For those wishing to go direct to Washington, all rail, the fare will be $12.50 to Washington, return on certificate at one-third fare.

For those who go to Washington first and would like to come back via. Old Point, we will sell tickets to Washington for $12.50 they pay their fare on the boat from Washington to Old Point and we will honor their certificates from Old Point to Cincinnati or Louisville at one-third fare.

We will arrange to stop over at the Springs for those who desire it. I am satisfied we can give your people a much pleasanter ride than any other line, and our rates and arrangements you will find are on as liberal a basis as it is possible to make them. I received a letter from Dr. Jordan, of St. Louis, and he says there is a probability that a Mineral Water Convention will be held at White Sulphur Springs after the meeting at Washington. Yours Respectfully,

D. G. EDWARDS, G. W. P. Agent.

We can recommend this as one of most pleasant and enjoyable routes to Washington, and indeed to all points East. The scenery is unsurpassed by that of any other route, and the service on the road we have rarely seen equalled and never surpassed. Those who have run the gauntlet of inattentive and surly officials will most surely appreciate this feature and rare commodity of the Chesapeake and Ohio route.

### Local Revulsive Action of Iodine.

If a piece of absorbent cotton wet with tincture of iodine be held in contact with the skin, any desired amount of revulsive effect may be obtained, even to blistering or the formation of an eschar.—*La Normandie Medicale.*

